# Effect of Manganese Alloying on Infrared Detectors Made of Pb_1−x_Mn_x_Te/CdTe Multilayer Composite

**DOI:** 10.3390/ma16124211

**Published:** 2023-06-06

**Authors:** Sergij Chusnutdinow, Alexander Kazakov, Rafał Jakieła, Michał Szot, Steffen Schreyeck, Karl Brunner, Grzegorz Karczewski

**Affiliations:** 1 Institute of Physics, Polish Academy of Sciences, aleja Lotników 32/46, 02-668 Warsaw, Poland; jakiela@ifpan.edu.pl (R.J.); szot@ifpan.edu.pl (M.S.); karcz@ifpan.edu.pl (G.K.); 2 International Research Center MagTop, Institute of Physics, Polish Academy of Sciences, aleja Lotników 32/46, 02-668 Warsaw, Poland; kazakov@magtop.ifpan.edu.pl; 3 Physikalisches Institut, Experimentelle Physik III, Universität Würzburg, D-97074 Würzburg, Germany; steffen.schreyeck@physik.uni-wuerzburg.de (S.S.); brunner@physik.uni-wuerzburg.de (K.B.)

**Keywords:** PbMnTe, CdTe, multilayers, infrared photodetectors, photoresistors, molecular beam epitaxy

## Abstract

The properties of Pb_1−x_Mn_x_Te/CdTe multilayer composite grown by molecular beam epitaxy on a GaAs substrate were studied. The study included morphological characterization by X-ray diffraction, scanning electron microscopy, secondary ion mass spectroscopy, as well as electron transport and optical spectroscopy measurements. The main focus of the study was on the sensing properties of photoresistors made of Pb_1−x_Mn_x_Te/CdTe in the infrared spectral region. It was shown that the presence of Mn in the Pb_1−x_Mn_x_Te conductive layers shifted the cut-off wavelength toward blue and weakened the spectral sensitivity of the photoresistors. The first effect was due to an increase in the energy gap of Pb_1−x_Mn_x_Te with an increase in Mn concentration, and the second was due to a pronounced deterioration in the crystal quality of the multilayers owing to the presence of Mn atoms, as shown by the morphological analysis.

## 1. Introduction

Infrared (IR) detectors are widely used for various purposes, such as chemical gas analysis, gas leak detection, IR imaging, remote temperature measurements, etc. In particular, there is continued interest in applications and consequently in the development of IR detectors operating at room temperature [1,2,3]. 

Structures composed of a combination of PbTe and CdTe have been the subject of study for quite some time and exhibit interesting and distinctive properties. In particular, the components are almost completely immiscible due to their different crystal structures, although they crystallize in cubic structures and have nearly identical lattice constants (6.46 and 6.48 Å, respectively). Specifically, PbTe has a rock salt structure, while CdTe has a zincblende structure. The PbTe/CdTe material system exhibits a crucial feature—a significant difference in energy gaps (PbTe = 0.32 eV; CdTe = 1.59 eV) and type I band ordering, which is supported by X-ray photoemission spectroscopy [4]. As a result, both background and photoexcited carriers are strongly localized and confined in low-dimensional PbTe objects, leading to efficient IR luminescence from PbTe/CdTe QDs and QWs [5,6]. By incorporating an array of PbTe QDs into a depletion layer of a CdTe *p*-*n* junction, researchers achieved the first demonstration of IR electroluminescence at room temperature [7]. 

The CdTe/PbTe heterojunction system, consisting of zincblende CdTe and rocksalt PbTe, was recently found to exhibit a two-dimensional electron gas (2DEG) at the (111) polar interface with exceptional carrier density and mobility. The 2DEG is attributed to the interfacial bonding coordination mismatch, which is different from traditional 2DEG systems [8,9,10,11]. The authors [12,13] showed that such a system was a good candidate for high-speed mid-infrared detectors.

In our previous publication, we showed that PbTe/CdTe multilayer composite was also an attractive candidate for IR detectors operating at high temperatures [14]. We demonstrated experimentally that photoresistors made of PbTe/CdTe multilayers were highly sensitive to infrared light, and their detectability at room temperature was comparable to that of commercially available infrared detectors [14]. 

The high performance of PbTe/CdTe detectors is due to three factors. The first factor is the significant reduction in the concentration of free carriers in conductive PbTe layers due to their capture by broken bonds located at the PbTe/CdTe interfaces. The atomic bonds at the interfaces are broken because of the different crystal structures of PbTe and CdTe, the rock salt and zincblende, respectively. The second factor is the high mobility of carriers present in the PbTe layers. Despite the huge number of defects at the interfaces, high mobility is preserved due to the high dielectric constant of PbTe, which effectively screens the scattering centers at the interface. The third factor is the reduction in Auger recombination. Auger recombination is the main factor limiting the detectability of devices made of narrow-bandgap semiconductors, especially at high temperatures. On the other hand, in wide-bandgap materials, such as GaAs [15] or CdTe [16], the much weaker Auger recombination plays a negligible role. In consequence, it has been proven experimentally that the Auger recombination can be significantly suppressed when the IR detectors are made of a combination of narrow- and wide-bandgap semiconductors. The effect was demonstrated for such material systems as InAs/Ga_x_In_1−x_Sb [17], HgTe/CdTe [18], Hg_x_Cd_1−x_Te/CdTe [19], PbSe/CdS [20], and recently, PbSe/CdTe [21]. The effective reduction in Auger recombination in PbSe/CdS and PbSe/CdTe devices resulted in a measurable spectral photoresponse up to room temperature even when the IR-sensitive single PbSe layer was very thin [20,21]. 

The morphology and thermally activated morphological transformations of PbTe/CdTe multilayers were shown experimentally and discussed in theoretical detail in previous publications [6,22,23].

The main objective of the present work was to determine how manganese atoms introduced into PbTe layers affected the performance of IR photoresistors made of Pb_1−x_Mn_x_Te/CdTe multilayer composite. It is known that Mn effectively increases the energy gap of Pb_1−x_Mn_x_Te [24], which leads to an increase in carrier effective mass and thus the density of states. 

## 2. Materials and Methods

### 2.1. Materials

In this work, we investigated structures comprising four Pb_1−x_Mn_x_Te/CdTe multilayers (MLs) grown by molecular beam epitaxy (MBE) on semi-insulating, (100) oriented GaAs substrate. The growth parameters, growth procedure, and thickness of the layers forming the structures were identical for all four samples except for the temperatures of the Mn cells used for alloying, which were 800, 820, and 850 °C, respectively. The last fourth investigated multilayer was undoped by Mn and it served as a reference. After the thermal removal of the oxide layer protecting the GaAs surface and cooling of the substrate in the Zn flux to the growth temperature of 240 °C, the Te shutter was opened for 30 s to deposit about 5 nm of ZnTe. The ultrathin ZnTe buffer layer was necessary to reduce the large lattice mismatch between GaAs and CdTe (13.6%) and further stabilize growth in the (100) orientation. A 0.6 µm CdTe buffer layer was then deposited for one hour. The bulk of the structures consisted of four Pb_1−x_Mn_x_Te layers deposited for 300 s and separated by CdTe barriers deposited for 360 s. The structures were finally covered with a CdTe cap layer grown for 1260 s. Upon completion of growth, the samples were immediately cooled to room temperature. Molecular beams of elemental Pb, Cd, Te, and Mn were evaporated by standard effusion cells. The CdTe films were deposited under slight Cd overpressure with beam equivalent pressures (BEP) for Cd and Te of 1.1 × 10^−6^ and 1.0 × 10^−6^ mbar, respectively. To avoid precipitation of Pb, the growth of Pb_1−x_Mn_x_Te required Te-rich conditions, so the BEP for Pb was kept at a value of 6.8 × 10^−7^ mbar and for Mn, it was kept at the order of 10^−8^ mbar. The MBE process was monitored in situ by reflection high-energy electron diffraction (RHEED), which showed sharp, streaky patterns throughout the growth, indicating good-quality structures. 

The photoresistors were fabricated by cutting small rods from the grown structures and soldering indium contacts to the ends of the rods (Figure S1). To enhance the diffusion of indium and ensure electrical contact to all Pb_1−x_Mn_x_Te layers, the rods with indium contacts were annealed at 200 °C for 10 min in an air atmosphere.

### 2.2. Characterization and Measurements

After growth, the multilayer structures were characterized by high-resolution X-ray diffraction (HRXRD) using a Philips X’Pert MRD diffractometer (Amsterdam, The Netherlands). The resolution in the x-direction was limited to about 6 arc sec by the instrumental broadening of the 4 Ge(220) monochromator and the acceptance angle of the analyzer crystal was 12 arc s.

Further morphological examination of the multilayer composites was performed by field emission scanning electron microscopy (FE-SEM) using a neon 40-Auriga Carl Zeiss microscope (Carl Zeiss AG, Jena, Germany).

Depth profiles were established using the SIMS technique with a CAMECA IMS6F micro-analyzer. The SIMS measurement was performed with cesium (Cs+) primary beam at an energy of 5.5 keV with the current kept at 7 nA. The size of the raster was about 150 × 150 µm^2^ and the secondary ions were collected from a central region of approximately 60 µm diameter. Secondary ions ^55^Mn^133^Cs+, ^114^Cd^133^Cs+, ^130^Te^133^Cs+, and ^208^Pb^133^Cs+ were collected by the electron multiplier. 

The optical spectra were measured using an infrared spectrometer consisting of an M150 Solar Laser System monochromator (Minsk, Belarus), a Newport 6363 IR light source (Newport Corporation, Irvine, CA, USA), and a Thorlabs variable chopper frequency mechanical chopper (Newton, USA). The resistors were biased with a DC voltage of 10 V. The photoinduced AC signal was amplified with a DLPCA-200 low-noise current amplifier (FEMTO Messtechnik GmbH, Berlin, Germany) and measured using a Zurich Instruments MLFI 5 MHz lock-in amplifier (Zurich, Switzerland). The measurements were performed at a frequency of 730 Hz. The MLFI lock-in amplifier also was used for measurements of noise power spectral density, which was needed to calculate the detectivity of the ML resistors.

The PL was excited by the radiation at 1064 nm emitted by a diode-pumped passively Q-switched solid state laser (DPSSL) with a single pulse duration of 1.3 ns and repetition frequency of 1 kHz. The energy of laser excitation was 1.17 eV, i.e., above the bandgap of PbTe but below the bandgap of CdTe. A HgCdTe infrared diode detector was used to detect the infrared PL signal in the wavelength range from 2 to 6 μm.

For the electron transport measurements, the samples were cleaved to the form of 2 × 6 mm rectangles. Six indium contacts in a Hall bar-like configuration were soldered from the top. A standard, low-frequency lock-in technique was applied for the resistance measurements. Measurements were performed until the contacts became non-Ohmic at lower temperatures.

## 3. Results

**Figure 1:**
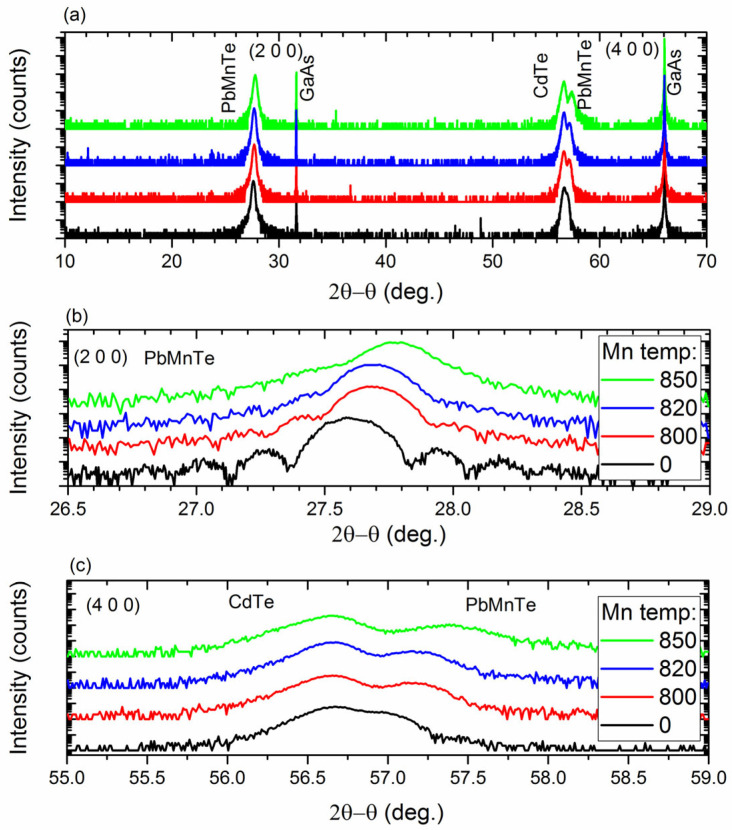
(**a**) The (2θ−θ) wide-angle X-ray diffraction scans of Pb_1−x_Mn_x_Te/CdTe multilayers and (**b**,**c**) the high-resolution diffraction scans.

The (2θ−θ) wide-angle XRD scans of all samples showed sharp and intense (200) and (400) peaks of Pb_1−x_Mn_x_Te and the GaAs substrate and the (400) peak of CdTe, as shown in Figure 1a. The (200) diffraction peak of the CdTe layer was hardly visible because of the structure factor. The absence of other peaks confirmed that the structures were (100) oriented and free of precipitation and other phases. The high-resolution diffraction scans presented in Figure 1b,c showed that the (200) peak of Pb_1−x_Mn_x_Te shifted toward higher angles with increasing Mn content, indicating a decrease in the lattice constant. Based on the angular positions of the HRXRD peaks and the relation a(x)= (6.46 − 0.632x) Å [25,26], one could estimate the Mn content, x, in the Pb_1−x_Mn_x_Te layers; x = 0.030, 0.032, and 0.063 for structures grown with the Mn source at 800, 820, and 850 °C, respectively. 

**Figure 2:**
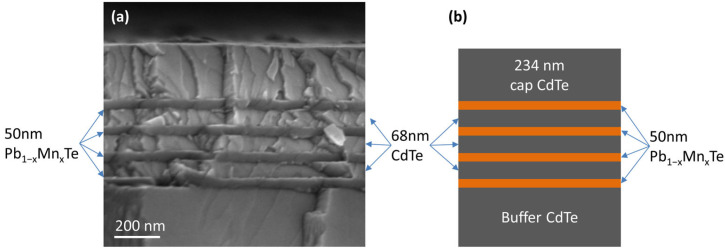
(**a**) SEM image of cleaved surface of Pb_1−x_Mn_x_Te/CdTe multilayers and (**b**) the schematic of the grown structure.

Due to the different easy cleavage planes of the crystal structures of CdTe and PbTe (zincblende and rocksalt, respectively), the SEM images showed high contrast between the layers forming the structures, as shown on the cleaved surface in Figure 2a. From the SEM data, the thicknesses of the layers forming the structures could be determined (Figure 2b): CdTe buffer 668 nm, Pb_1−x_Mn_x_Te layers 50 nm, CdTe barrier 68 nm, CdTe cap 234 nm. These results indicated that for the applied fluxes and substrate temperature of 240 °C, the growth rates of CdTe and Pb_1−x_Mn_x_Te were about 0.19 and 0.17 nm/s, respectively.

The multilayer structures, as shown in Figure 2, were used to fabricate simple photoresistors. By cleaving the as-grown wafer, 1 × 3 mm bars were obtained. To create electrical contacts at both ends of the bars, small pieces of indium were soldered to the top CdTe cap. The In-contacted bars were then annealed at 200 °C for 10 min in an air atmosphere. The annealing resulted in diffusion of In into the sample, ensuring good electrical contact with all PbTe layers. The current–voltage characteristics are shown in Figure S2.

Spectral responsivity, *R_i_*, i.e., the ratio of the detector output signal to the incident optical power, was determined for samples with various Mn contents at 77 and 300 K. The incident optical power, *P*, for each wavelength was first measured using a Vigo PVMI-4TE-8 calibrated detector (Ożarów Mazowiecki, Poland). After measuring the output current signal, *I_ph_*, the spectral responsivity was determined from the relation:

(1) $$R_{i}=I_{ph}/P\;(A/W)$$

**Figure 3:**
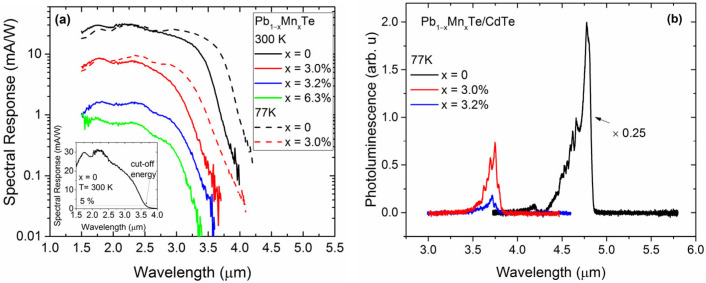
(**a**) Spectral response of the Pb_1−x_Mn_x_Te/CdTe photoresistors at 77 and 300 K. In the inset, the spectral response of the PbTe/CdTe multilayer photoresistor on a linear scale. (**b**) Photoluminescence spectra of the Pb_1−x_Mn_x_Te/CdTe structures at 77 K.

As shown in Figure 3a, the Pb_1−x_Mn_x_Te/CdTe multilayer photoresistors showed pronounced current responsivity in the infrared spectral region. The temperature change had a rather little effect on the intensity of the photoresponse signal, especially at higher spectral energies. Such an unexpected effect was also observed for PbTe/CdTe detectors [4]. On the other hand, lowering the measurement temperature shifted the cut-off energy of the photoresponse spectrum (the cut-off energy was arbitrarily defined as the longest wavelength at which detector responsivity reached 5% of the peak value, illustrated in the inset of Figure 3a) toward lower energies, reflecting a temperature dependence of the energy bandgap of Pb_1−x_Mn_x_Te.

The presence of Mn atoms in the structures affected the photoresponse spectra much more than the temperature. It shifted the high-energy cut-off toward higher energies and caused a significant decrease, by more than one order of magnitude, in the responsivity across the entire spectral range. The observed blue shift in the cut-off energy was due to an increase in the energy gap with increasing Mn concentration in the Pb_1−x_Mn_x_Te layers. The possible reasons for the decrease in the optical responsivity will be discussed below. 

The spectral responsivity was used to calculate the specific detectivity. The specific detectivity of the photoresistor, D*, was calculated using the well-known relationship:

(2) $$D^{*}=\frac{R_{i}R\sqrt{A\Delta f}}{V_{n}}=\frac{R_{i}R\sqrt{A}}{S_{n}}\;(\mathrm{cm}\;\times \;{\mathrm{Hz}}^{1/2}/W)$$

where *A* is the photosensitive surface area of the detector; *R_i_* is the spectral responsivity expressed in *A*/*W*; Δ*f* is the frequency bandwidth; *S_n_* is the noise voltage spectral density expressed in units of *V*/Hz^1/2^, which was measured using an MLFI lock-in amplifier; and *R* is the resistance of the structure determined from the I–V characteristic (Figure S2) according to the formula *R* = (*dV*/*dI*)*_V_*_=0_. The specific detectivity was calculated for the 2.5 µm wavelength at 300 K. The parameters used to calculate the specific detectivity and its values are summarized in Table 1. 

As seen in Figure 3a and Table 1, the spectral responsivity and specific detectivity decreased strongly with increasing Mn content. In order to determine the reasons for the decrease, the multilayer structure was characterized by photoluminescence (PL), electron transport measurements, and secondary ion mass spectroscopy (SIMS) to verify the real profile of atomic distribution in the structure. 

The PL spectra shown in Figure 3b showed that even for samples with relatively low Mn content (x = 0.03 and x = 0.032), the PL signal intensity decreased by an order of magnitude. For a structure with x = 0.063, the PL signal disappeared completely. This effect was probably due to the formation of defects in the Pb_1−x_Mn_x_Te layers. The atomic radius of Mn is much smaller than that of Pb or Te, so the incorporation of Mn into the crystal lattice caused local deformation and distortion of the lattice. In addition, the solubility of Mn in PbTe is limited. In bulk PbTe, the reported solubility of Mn is approximately 10–12%. Above this value, MnTe and MnTe2 inclusions occur [27]. Despite the fact that the structures studied contained less Mn and we did not observe any foreign phases in our structures, it is very likely that the presence of Mn in PbTe caused the formation of defects, both point and extended. At least some of them may have acted as non-radiative recombination centers that kill the PL signal.

**Figure 4:**
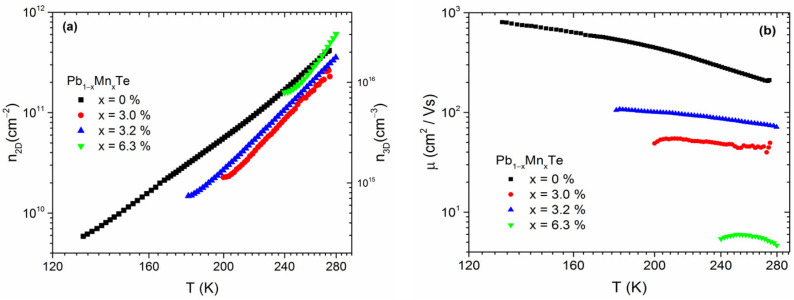
(**a**) Electron carrier concentration and (**b**) electron mobility of the Pb_1−x_Mn_x_Te/CdTe multilayer structure plotted in logarithmic scale.

The temperature dependencies of the carrier concentration, *n*, and carrier mobility, µ, are shown in Figure 4a,b, respectively. To estimate the concentration of the 3D carrier, we assumed a cumulative conductor thickness of 200 nm. The structures showed *n*-type conductivity. The type of conductivity in undoped IV–VI compounds depends mainly on stoichiometry. Adjusting the flux ratio between metal and chalcogen atoms during growth allows the formation of crystals with Pb vacancies that act as double-charged acceptors and Te vacancies that act as double-charged donors. Typically, Pb_1−x_Mn_x_Te bulk crystals are *p*-type [24,28], but there are reports of Pb_1−x_Mn_x_Te-based nanostructures that are *n*-type [29].

In the studied structures, the Mn atoms did not significantly affect the concentration of carriers. At high temperatures, the electron concentrations of all structures were of the same order of magnitude, i.e., 10^11^ cm^−2^. With decreasing temperature, the concentration of electrons decreased because of the freeze-out effect. On the other hand, the electron mobility did not depend much on the temperature in the studied temperature range, but it strongly decreased with increasing Mn content. In the case of bulk Pb_1−x_Mn_x_Te, the carrier mobility also depended on the Mn content [25]. This can be explained by the fact that the presence of Mn atoms in the lattice introduced disorder and thus enhanced the scattering of mobile charge carriers, which led to a decrease in mobility with increasing x. 

**Figure 5:**
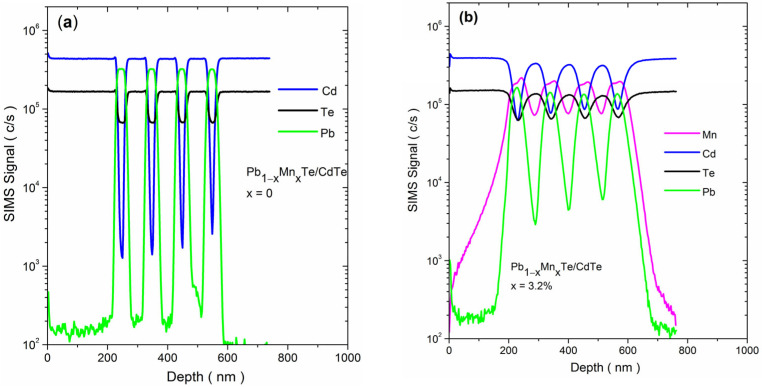
SIMS depth profile of Mn, Cd, Te, and Pb in the Pb_1−x_Mn_x_Te/CdTe multilayers photoresistors, (**a**) x = 0 and (**b**) x = 3.2%.

Another possible reason for the decrease in mobility and weakening of the intensity of the photoresponse with increasing Mn content was revealed by secondary ion mass spectroscopy (SIMS). The profiles of atomic distribution for Cd, Pb, and Mn measured by SIMS in the reference multilayer and those with x = 3.2% are compared in Figure 5a,b. While the distribution profiles of Cd and Pb atoms in the reference structure were sharp, indicating abrupt interfaces between PbTe and CdTe layers, the profiles of multilayers containing Mn were blurry. In particular, the atomic distribution of Mn atoms in the multilayers was much broader than that in pure PbTe layers, indicating a significant diffusion of Mn into the neighboring CdTe layers. This indicated that alloying with Mn atoms significantly lowered the morphological quality of PbTe/CdTe multilayers and thus their spectral sensitivity. This result also agreed with the damping of XRD fringes with increasing Mn content, as shown in Figure 1b. Based on the distribution profile of Mn atoms in the PbTe layers, it appeared that the distribution of Mn was not uniform. There was a slightly higher concentration of Mn atoms at the interface between PbTe and CdTe layers. This suggested that Mn may have been segregating on these interfaces.

## 4. Conclusions

In summary, we studied the effects of Mn alloying on the properties of a Pb_1−x_Mn_x_Te/CdTe multilayer composite grown by molecular beam epitaxy on GaAs. The study included high-resolution X-ray diffraction morphological characterization by scanning electron microscopy, secondary ion mass spectroscopy, magneto-transport, and optical properties analysis. The results showed that Mn was incorporated into the PbTe layers and partially diffused into the CdTe layers. The incorporation of Mn increased the interface roughness and lattice mismatch between the rocksalt and zincblende structures. Formed distortions could result in the creation of defects, acting, at least partially, as centers of non-radiative recombination and thus decreasing the optical effects. The decrease in PL intensity and total disappearance of structures containing 6.3 at.% Mn confirmed the creation of such non-radiative recombination centers. The main focus of the study was on photoresistors made of Pb_1−x_Mn_x_Te/CdTe and their sensing properties in the infrared spectral region. It was shown that the presence of Mn in the conductive layers of Pb_1−x_Mn_x_Te weakened the spectral sensitivity and shifted its cut-off at long wavelengths toward blue. The first effect was due to an increase in the energy gap of Pb_1−x_Mn_x_Te with an increase in Mn concentration, and the second effect was due to a pronounced deterioration in the crystal quality of the multilayers owing to the presence of Mn atoms. The specific detectivity was calculated. 

## Figures and Tables

**Table 1:** Measured and calculated parameters of photodetector.

Mn Content	$R_{i}$	$S_{n}$	*R*	A	$D^{*}$
%	mA/W	$\frac{V}{\sqrt{\mathrm{Hz}}}$	kOhm	${\mathrm{cm}}^{2}$	$\mathrm{cm}\times \frac{\sqrt{\mathrm{Hz}}}{W}$
0	25.0	1.1 × 10^−8^	7.5	0.021	3.5 × 10^8^
3.0	6.3	1.6 × 10^−8^	17.0	0.025	1.7 × 10^8^
3.2	1.4	1.5 × 10^−8^	14.2	0.028	3.6 × 10^7^
6.3	0.60	2.1 × 10^−8^	8.6	0.019	8.0 × 10^6^

## Data Availability

Data are available through email upon a reasonable request.

## Supplementary Materials

The following supporting information can be downloaded at: https://www.mdpi.com/article/10.3390/ma16124211/s1, Figure S1: Picture of the fabricated typical photoresistor, Figure S2: Current–voltage characteristics of fabricated photoresistors.
