# Supplementary material for: Effect of Manganese Alloying on Infrared Detectors Made of Pb1−xMnxTe/CdTe Multilayer Composite
Source: Materials (Basel). 2023 Jun 6;16(12):4211. doi: 10.3390/ma16124211 (PMC10304039; doi:10.3390/ma16124211)
Supplement: Supplementary file 1 [file materials-16-04211-s001.zip › materials-2340958-supplementary.pdf]

Supplementary Information

Effect of Manganese Alloying on Infrared Detectors Made of $Pb_{1-x}Mn_xTe$/CdTe Multilayer Composite

Sergij Chusnutdinow [1,2,*], Alexander Kazakov [2], Rafał Jakieła [1], Michał Szot [1,2], Steffen Schreyeck [3], Karl Brunner [3] and Grzegorz Karczewski [1]

[1] Institute of Physics, Polish Academy of Sciences, aleja Lotników 32/46, 02-668 Warsaw, Poland; jakiela@ifpan.edu.pl (R.J.); szot@ifpan.edu.pl (M.S.); karcz@ifpan.edu.pl (G.K.)
[2] International Research Center MagTop, Institute of Physics, Polish Academy of Sciences, aleja Lotników 32/46, 02-668 Warsaw, Poland; kazakov@magtop.ifpan.edu.pl
[3] Physikalisches Institut, Experimentelle Physik III, Universität Würzburg, D-97074 Würzburg, Germany; steffen.schreyeck@physik.uni-wuerzburg.de (S.S.); brunner@physik.uni-wuerzburg.de (K.B.)
* Correspondence: chusnut@ifpan.edu.pl

Figure

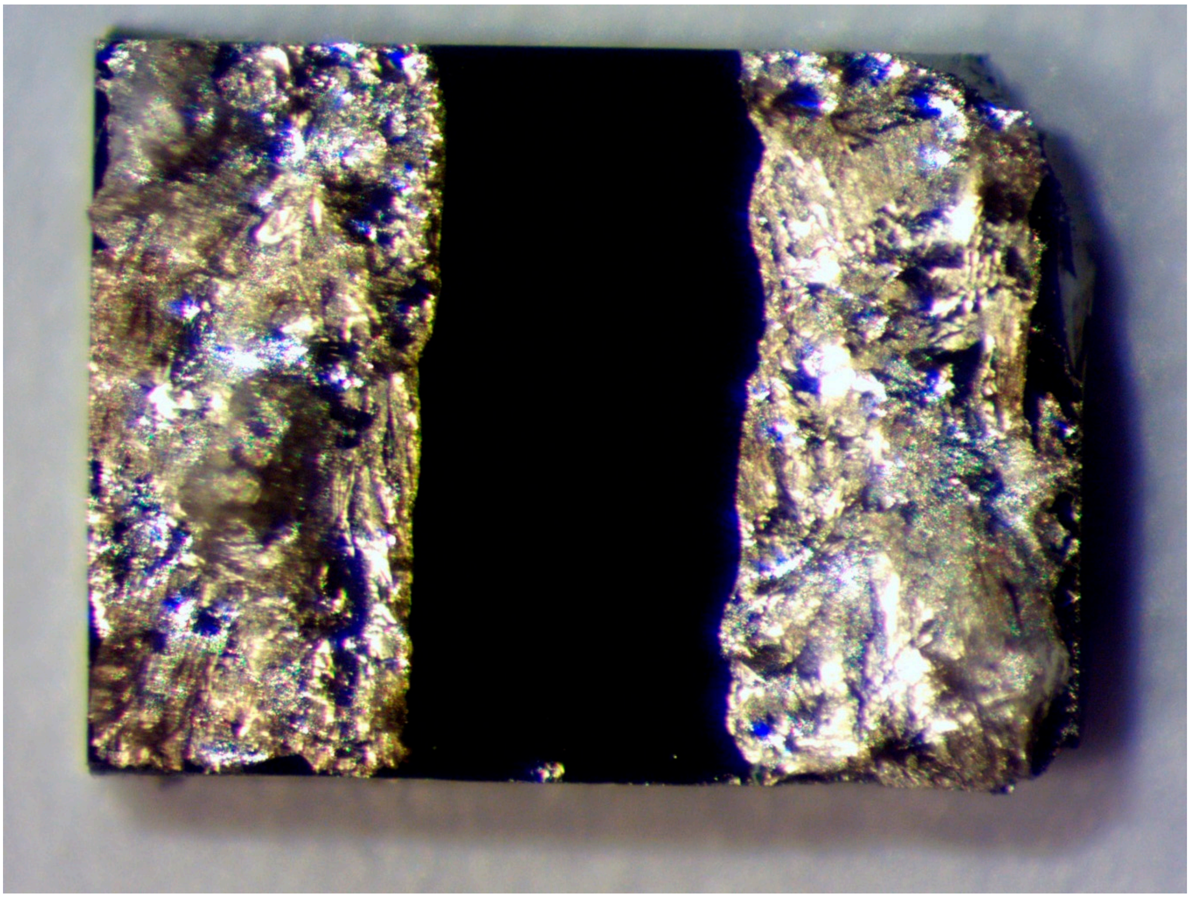

Figure S1. Picture of the fabricated typical photoresistor

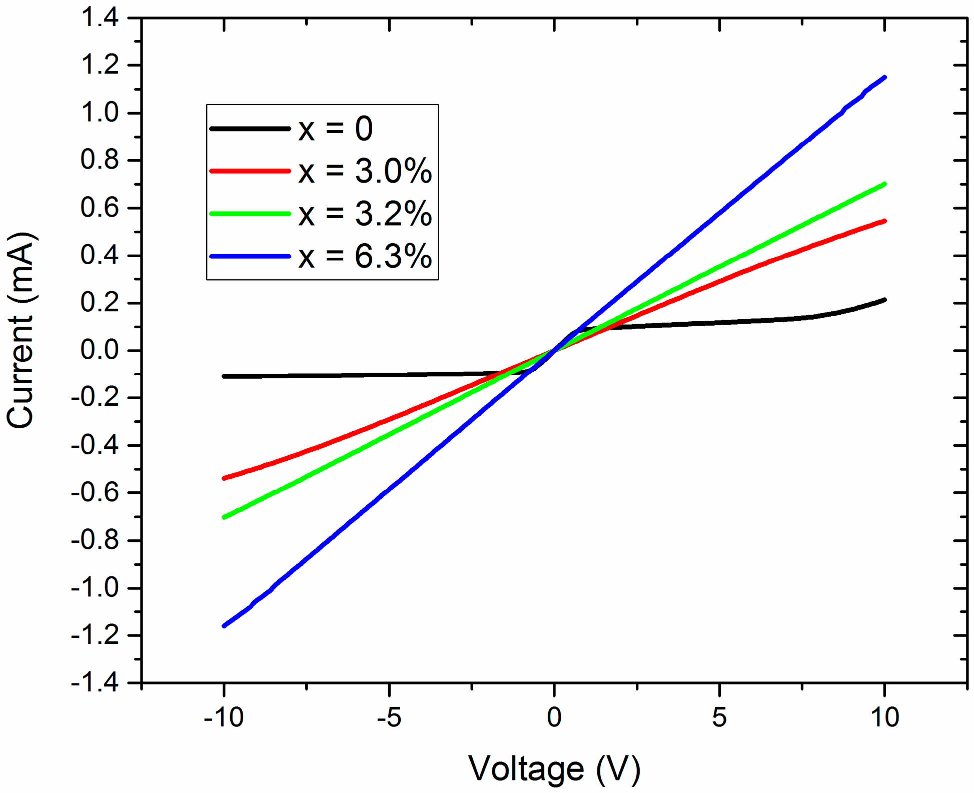

Figure S2. Current-voltage characteristics of fabricated photoresistors
